# Supplementary material for: Discovery and characterization of a new type of domain wall in a row-wise antiferromagnet
Source: Nat Commun. 2021 Jun 9;12:3488. doi: 10.1038/s41467-021-23760-2 (PMC8190316; doi:10.1038/s41467-021-23760-2)
Supplement: Supplementary file 1 — Supplementary Information [file 41467_2021_23760_MOESM1_ESM.pdf]

Supplemental Material: Discovery and characterization of a new type of domain wall in a row-wise antiferromagnet

Jonas Spethmann,[1,*] Martin Grünebohm,[1] Roland Wiesendanger,[1] Kirsten von Bergmann,[1] and André Kubetzka[1,†]

[1]*Department of Physics, University of Hamburg, 20355 Hamburg, Germany*

(Dated: March 24, 2021)

S1: Electronic Contrast of Domain Walls
S2: Phase Domain Walls
S3: Domain Walls, Crystal Anisotropy and ASE
S4: Fourth-Order HOI Terms across Domain Walls
S5: Spin Textures and Profiles of Simulated Domain Walls
S6: Influence of Biquadratic Interaction on Domain Walls
S7: Domain Wall Width and Energy
S8: Domain Walls in a Checkerboard AFM on a Square Lattice

S1: Electronic Contrast of Domain Walls

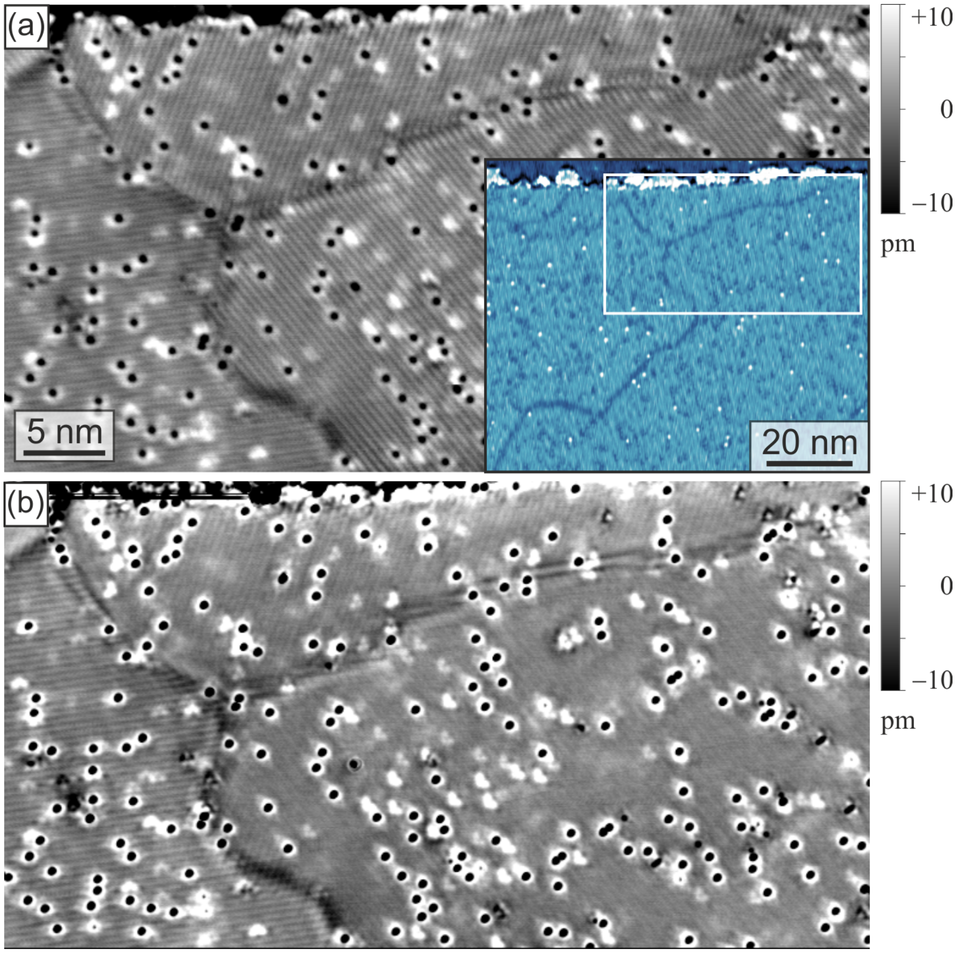

Figure S1. (a) SP-STM image of three $1Q$ rotational domains, as shown in Fig. 1. (b) The same area as in (a) recorded after a controlled tip-surface collision ($U = -30$ mV, $I = 7$ nA, Cr tip, $T = 4.2$ K). The magnetic corrugation has decreased significantly for two rotational domains, but specific patterns of the DWs remain clearly visible. This indicates a sizable influence of the DW onto the spin-averaged local density of states (LDOS), which allows detection of DWs at larger scales, as shown in the inset (dI/dU map, $U = -30$ mV, $I = 7$ nA), without requiring spin-sensitivity or atomic scale resolution. The LDOS variations at the DWs are probably in part caused by a mechanism similar to the non-collinear magnetoresistance (NCMR) [1]. The NCMR has its origin in the mixing of bands of majority and minority spin channels that occurs in non-collinear spin structures and is therefore highly dependent on the local spin environment, i.e., the angle between adjacent spins. In principal, tunneling anisotropic magnetoresistance (TAMR) [2] could be partly responsible for the DW LDOS variations, too. The TAMR is a material specific effect caused by spin-orbit coupling and depends on the local sample magnetization direction. However, for DWs in which the spin quantization axis remains fully in the surface plane, we expect only a small contribution by TAMR, due to the relatively isotropic in-plane directions of the system [3].

S2: Phase Domain Walls

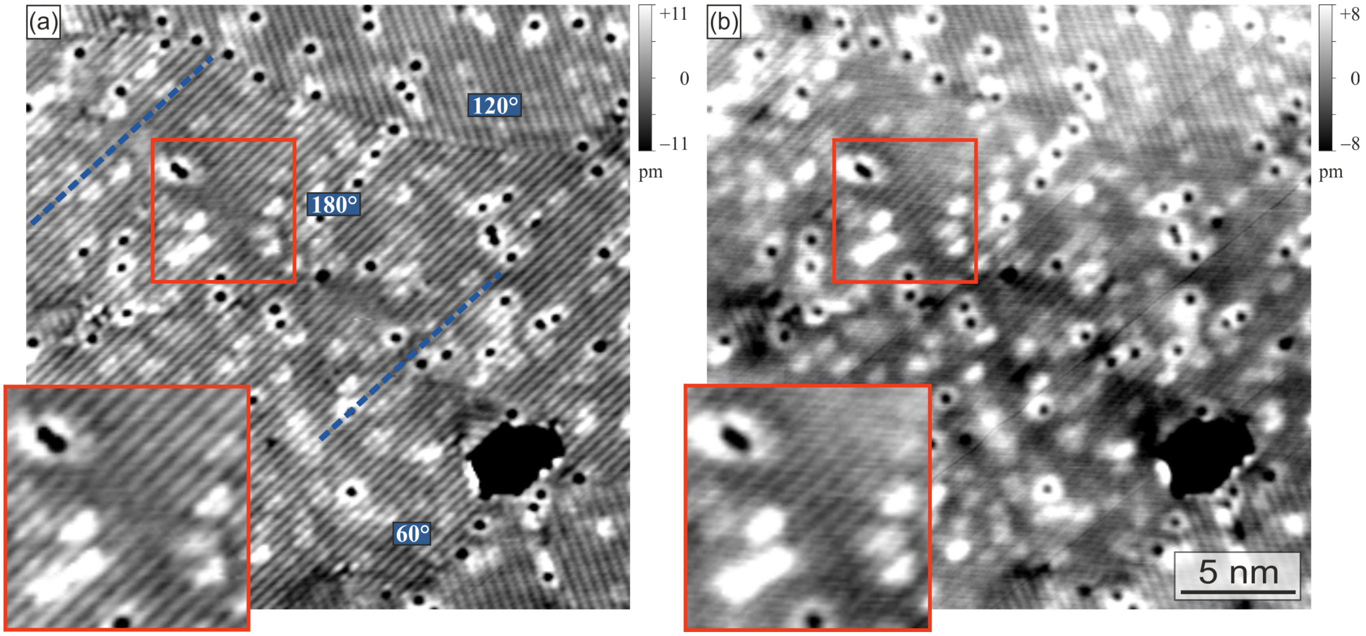

Figure S2. In addition to the 60° and 120° domain walls (DWs), on rare occasions we observe a third wall type with a spin rotation of 180°. Unlike DWs that occur at the boundary of two rotational domains, the 180° wall exists between two phase inverted domains with antiferromagnetic (AFM) rows along the same direction. Such a phase DW runs diagonally through the central row-wise AFM (1Q) domain shown in (a) and (b), starting at the upper left corner and ending at the vacancy island (black; $U = -20$ mV, $I = 7.5$ nA, $B = 0$ T, Fe/W tip, $T = 8$ K). In (a) the phase DW is visible as a shift in the AFM contrast along a narrow path. The dashed lines are aligned parallel to the AFM stripes and serve as visual guides. See inset for a magnified view of the DW ($B = 0$ T). (b) shows the same area, but the tip magnetization direction has changed after a gentle tip-surface collision and an external magnetic field has been applied perpendicular to the sample surface ($B = 2$ T). Here the AFM contrast almost vanishes on the central rotational domain, i.e., the tip magnetization axis is nearly perpendicular to the AFM rows, but the phase DW shows a strong AFM contrast. From this data we conclude that the 180° phase domain wall is realized as a uniform rotation of the spin quantization axis equivalent to the AFM phase DWs found in Fe/W(001) [4].

S3: Domain Walls, Crystal Anisotropy and ASE

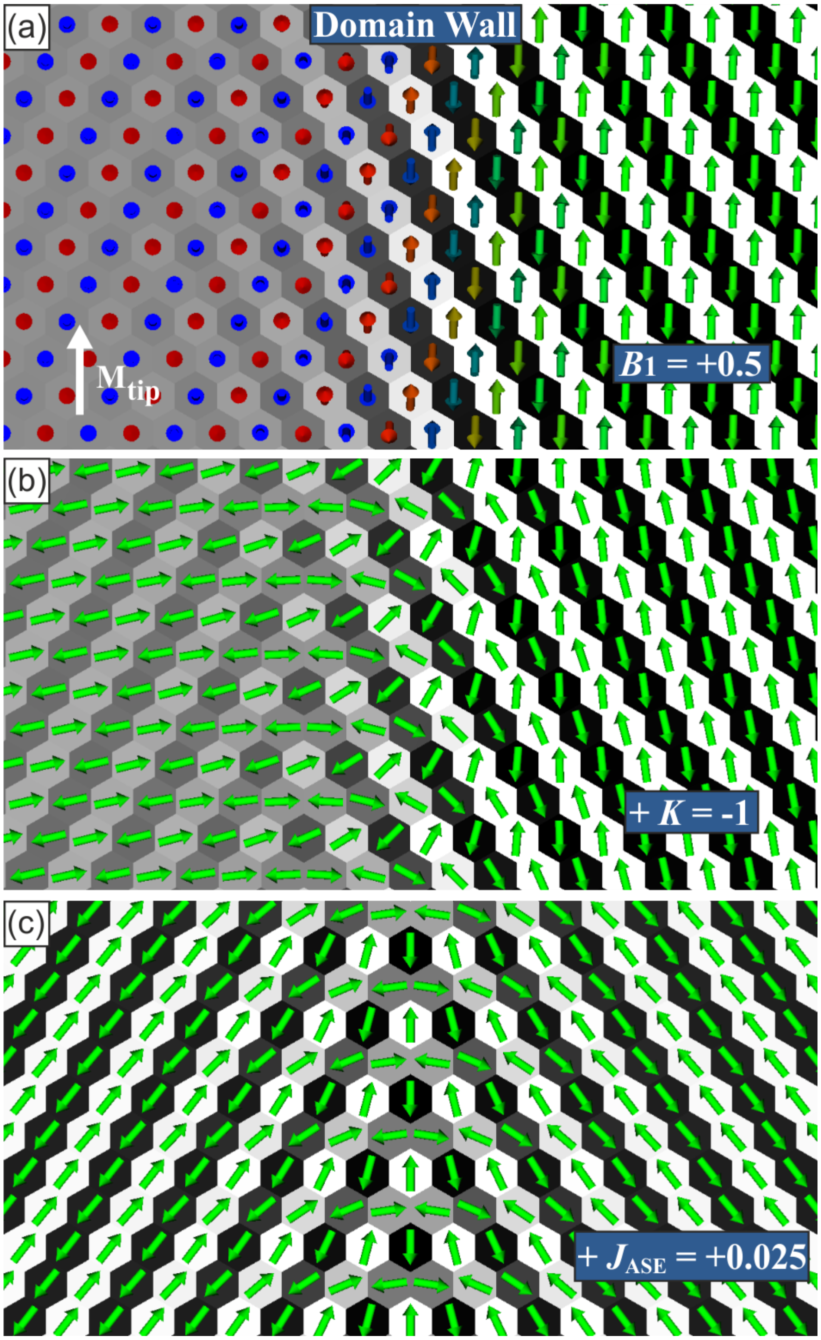

Figure S3. Atomistic spin dynamics simulations [5] of two 1Q domains and a 60° DW with step-wise more interactions considered during the simulations. Red and blue arrows are pointing in opposite out-of-plane directions, green arrows are in-plane. The simulations were performed in a 40 x 90 hexagonal grid with open boundaries using a LLG algorithm [5]. (a) $J_1 = -25$, $J_2 = -5$, $B1 = +0.5$: this results in two 1Q domains with spin quantization axes that are not coupled to the crystal lattice in any way. A transient 2Q state is formed in the center of the wall and induces a 90° spin rotation between the adjacent domains. (b) An easy-plane anisotropy $K = -1$ rotates all spins towards the surface plane. (c) With the inclusion of asymmetric exchange interaction $J_{\rm ASE} = +0.025$ the spin quantization axis is now coupled to the AFM row direction, however, close to a DW the spins are not perfectly aligned with the rows. This is caused by the conflict between the 2Q state in the DW and the 1Q domains, that demand a 60° or 120° rotation across the domain wall. All parameters in meV/atom. The DWs were generated by repeated cool downs from random spin configurations.

S4: Fourth-Order HOI Terms across Domain Walls

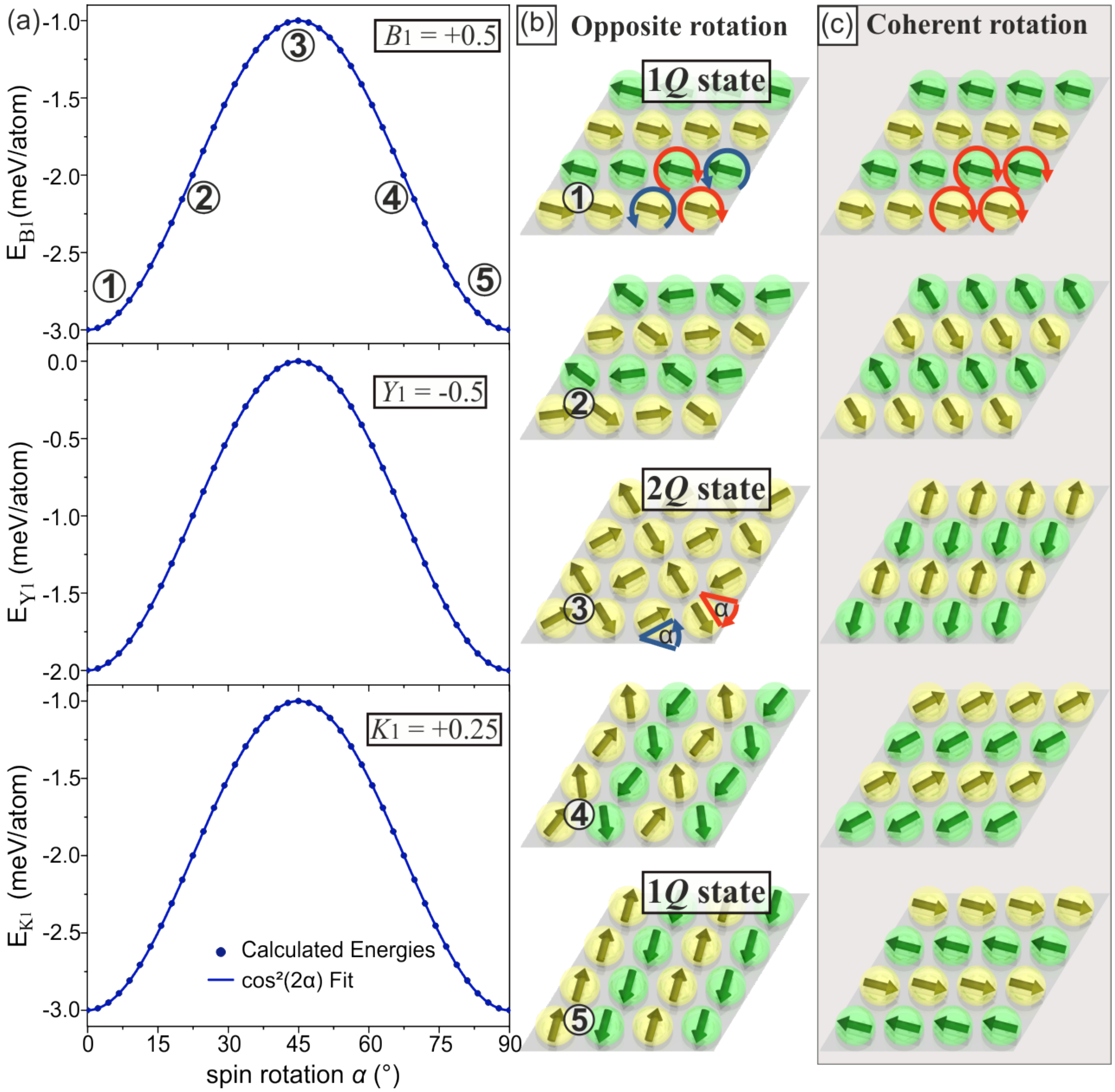

Figure S4. (a) Energy contribution of the three fourth-order HOI terms in a 1Q-2Q-1Q transformation, which is comparable to their energy contribution across a DW between two rotational domains. The transformation is realized by rotating specific spin pairs in opposite directions with an angle α from 0° to 90° [6, 7]. Blue dots are calculated energies for different spin configurations. All HOIs show the same $\cos^2(2\alpha)$ dependency and the data was fitted by using the function $E(\phi) = y + a \cdot \cos^2(2\alpha)$. The sketches in (b) show the pathway of the transformation, starting and ending at different rotational domains of the 1Q state, across a 2Q state at $\alpha = 45°$. The resulting rotational domain depends on the choice of the specific pairs of spins that are rotated in opposite directions. All spins are fully in-plane and rotated around the z-axis. The spins start with an angle of 15° away from the close-packed rows to fully reflect the 90° nature of a DW (see Fig. S3). (c) shows a uniform rotation of the spin quantization axis, as it is expected for a 180° phase DW (see Fig. S2), which does not rotate the AFM rows.

S5: Spin Textures and Profiles of Simulated Domain Walls

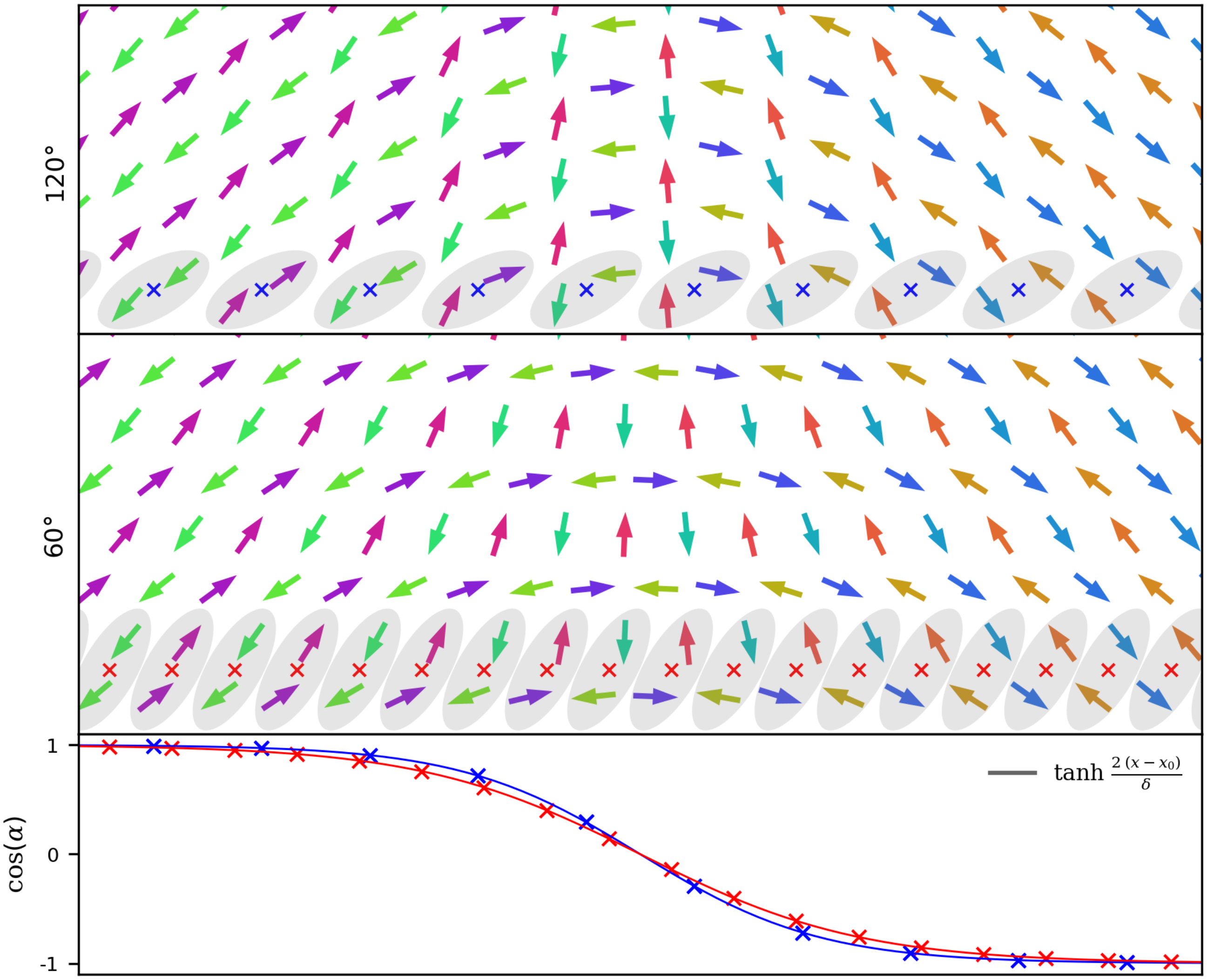

Figure S5. Spin configuration and wall profile of a simulated 120° and 60° DW. Color coding represents the azimuthal angle of the magnetic moments. The DW profile can be quantified as the angle $\alpha(x)$, enclosed by pairs of adjacent spins (marked by gray shading), in the direction perpendicular to the DW. Due to the different rotational 1Q domains left and right of the DW, a parallel pair in one domain will have an anti-parallel counterpart on the other side of the DW and a perpendicular orientation in the DW center. In close resemblance to the rotation of the quantization axis in FM DWs, $\cos(\alpha(x))$ can be described by $\tanh(2(x - x_0)/\delta)$, where δ is the DW width and $x - x_0$ the distance to the DW center. The presented configurations are the results of simulations, numerically integrating the LLG equation on a 1000×8 lattice, with open boundary conditions at the left and right edges and periodic boundary conditions along the DW. Starting from a configuration of two domains without transient region, the system was relaxed until the resulting δ converged to the sixth significant digit. The interaction parameters used here closely follow the DFT results for fcc Mn/Re(0001) [3], whereas B_1 is chosen as an effective HOI term in accordance with the observed DW widths: $J_1 = -24.7$, $J_2 = -5.6$, $B_1 = +0.5$ and $K = -1$, all in meV/atom.

S6: Influence of Biquadratic Interaction on Domain Walls

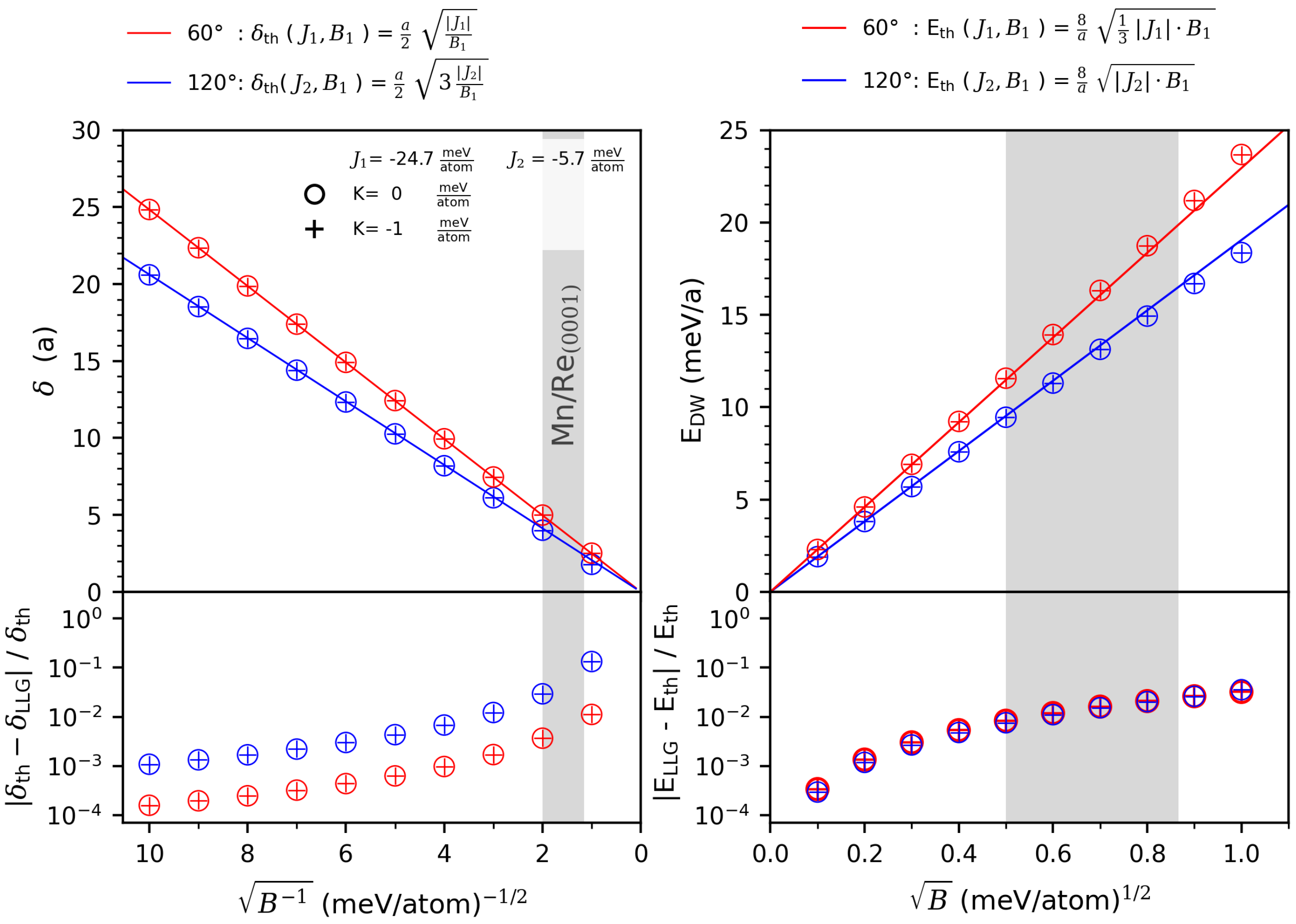

Figure S6. DW width δ (left) and DW energy E_{DW}(right) as a function of B_1, comparing the analytical formulas with numerical calculations, with and without uniaxial crystal anisotropy of the form $-K(S_z)^2$. For both types of DWs, 60° (red) and 120° (blue), the numerical results for $K = 0$ (circles) are in very good agreement with the analytical formulas presented in the main text. Including an easy-plane anisotropy, $K = -1$ meV/atom, into the calculation (crosses) does not lead to observable changes in energy or width, but enforces a coplanar spin texture, see Figs. S3(a),(b). For increasing strength of the biquadratic interaction B_1, the energy cost for DW formation increases as a consequence of the growing energy difference between the transient $2Q$ state and the favored $1Q$ domains. At the same time, the increasing contribution of B_1 leads to a decrease of DW width. For the range of B_1 (effective HOI strength) compatible with the experiment (gray area), the deviations between numerical results and analytical formulas are on the order of 0.5% and 3% for the 60° and 120° DW, respectively. The relative error in energy is on the order of 1% for both DWs and scales with the inverse DW width, indicating that these deviations are due to the small angle approximation the analytical formulas are based on.

S7: Domain Wall Width and Energy

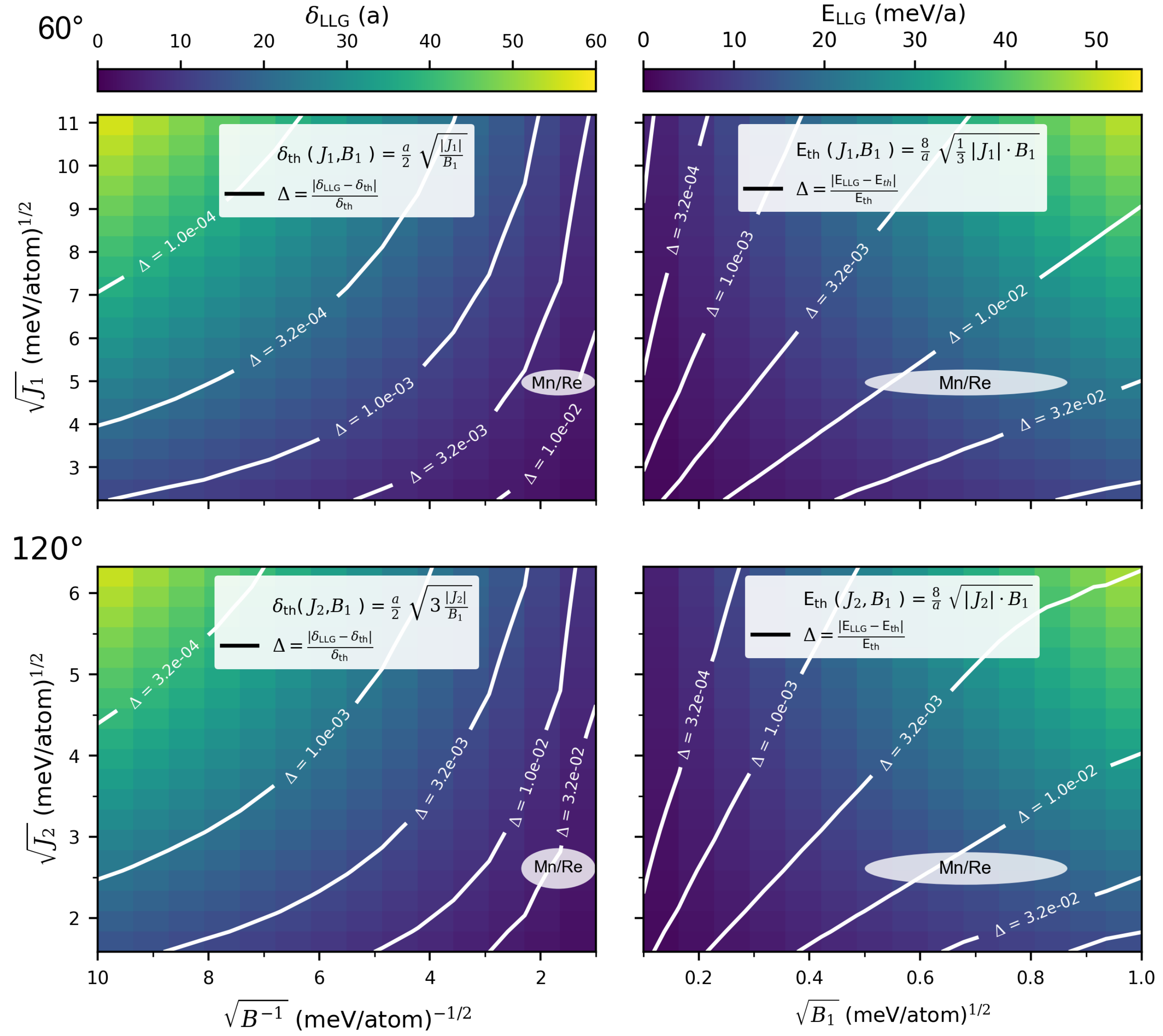

Figure S7. To further explore the interplay of the different interactions and to test the accuracy of the analytical formulas, a larger set of simulations was performed. The determined widths and energies for the 60° and 120° DWs are presented as color maps. Lines indicate the corresponding relative deviation between numerical and analytical result. For all values of J_1, J_2 the influence of the biquadratic interaction B_1 is as described in Fig. S6. As expected, the impact of varying J_1, J_2 on the DW width is opposite to that of B_1. For increasing exchange interaction the DW increases in width and energy. For small values of B_1, $B_1 \ll J_1, J_2$, the energy gap between $1Q$ and $2Q$ state closes, leading to wide DWs. In this regime, the difference between numerical result and theoretical prediction vanishes. The DW energy on the other hand increases for increasing values of B_1.

S8: Domain Walls in a Checkerboard AFM on a Square Lattice

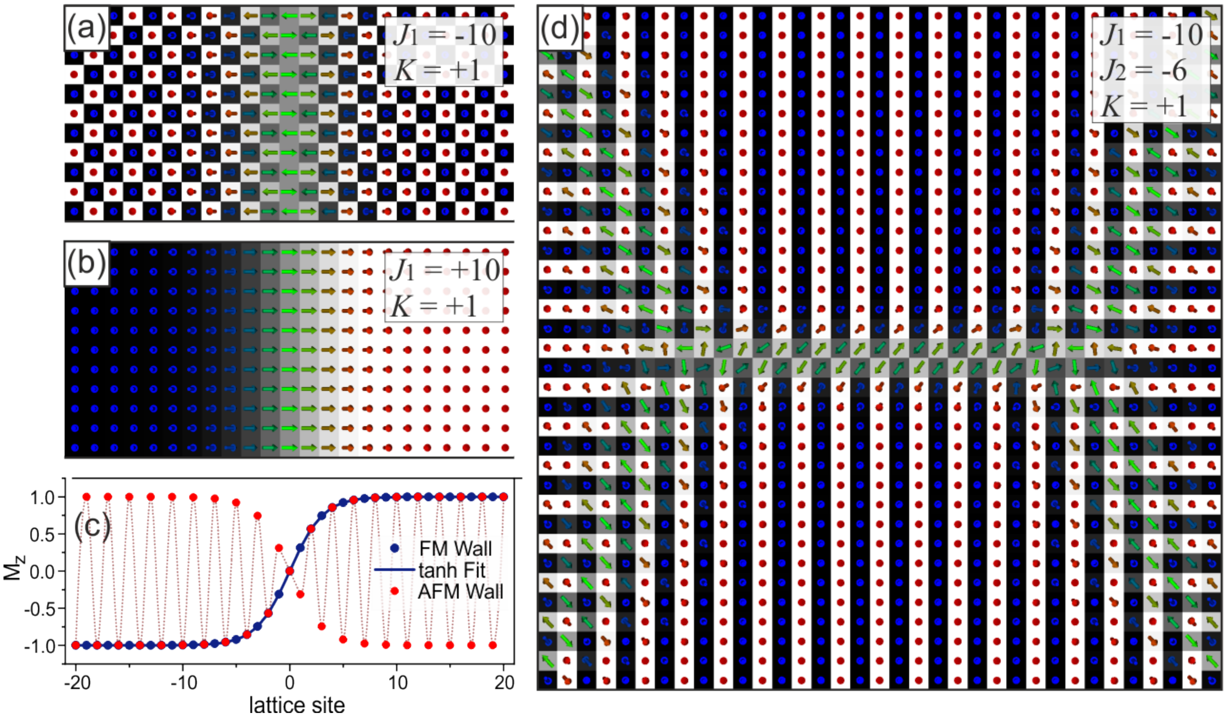

Figure S8. Phase domain walls and $2Q$ walls on a square lattice. (a) Phase DW in a checkerboard AFM stripe with out-of-plane easy axis, like in Fe/W(001)[4]. The DW configuration is metastable, relaxed via LLG energy minimization [5]. (b) When one sublattice is inverted and the sign of J_1 is changed from negative to positive, the resulting FM wall is already in its relaxed state. (c) Consequently, the DW profile of a sublattice in (a) is identical to the FM DW profile in (b). (d) When exchange frustration is introduced by a sufficiently strong J_2, the magnetic ground state changes to RW-AFM. In addition to the central phase DW, between rotational domains a new type of DW is possible, which again looks like a superposition state of two single spiral ($1Q$) states. Interestingly, the coupling to the edges leads to a metastable state, which is reminiscent of the Landau state of confined FM systems.

* Corresp. author: jspethma@physnet.uni-hamburg.de
† Corresp. author: kubetzka@physnet.uni-hamburg.de

[1] C. Hanneken, F. Otte, A. Kubetzka, B. Dupé, N. Romming, K. von Bergmann, R. Wiesendanger, and S. Heinze, Nat. Nanotech. **10**, 1039 (2015).
[2] M. Bode, S. Heinze, A. Kubetzka, O. Pietzsch, X. Nie, G. Bihlmayer, S. Blügel, and R. Wiesendanger, Phys. Rev. Lett. **89**, 237205 (2002).
[3] J. Spethmann, S. Meyer, K. von Bergmann, R. Wiesendanger, S. Heinze, and A. Kubetzka, Phys. Rev. Lett. **124**, 227203 (2020).
[4] M. Bode, E. Y. Vedmedenko, K. von Bergmann, A. Kubetzka, P. Ferriani, S. Heinze, and R. Wiesendanger, Nat. Mater. **5**, 477 (2006).
[5] The simulation code is available at https://github.com/JHagemeister/MonteCrystal.
[6] P. Kurz, PhD thesis, Aachen, Germany (2000).
[7] S. Meyer, B. Dupé, P. Ferriani, and S. Heinze, Phys. Rev. B **96**, 094408 (2017).
